# Effects of age and sex on the content of heavy metals in the hair, liver and the *longissimus lumborum* muscle of roe deer *Capreolus capreolus* L.

**DOI:** 10.1007/s11356-021-16425-6

**Published:** 2021-09-16

**Authors:** Dorota Cygan-Szczegielniak, Karolina Stasiak

**Affiliations:** Department of Animal Physiology and Physiotherapy, Faculty of Animal Breeding and Biology, Bydgoszcz University of Science and Technology, Mazowiecka 28, 85-004 Bydgoszcz, Poland

**Keywords:** Roe deer, Heavy metals, Hair, Liver, Muscle, Sex and age

## Abstract

The aim of the study was to investigate the effects of age and sex on the content of heavy metals (Pb, Cd, Zn and Cu) in the hair, liver and the *longissimus lumborum* muscle of roe deer (*Capreolus capreolus* L.). In addition, correlations between heavy metals in individual tested matrices were studied as well as an attempt was made to explain the reasons for their accumulation in specific research matrices. The levels of Zn and Cu were measured using atomic absorption spectroscopy (F-AAS). The levels of Pb and Cd were analysed by means of electrothermal atomic absorption spectroscopy (ET-AAS). In the own research, sex-related statistically significant differences in concentrations of analysed elements were confirmed. Higher levels of the elements were observed in samples collected from does (except for Cd in the liver samples of 6–7-month-old male fawns) but only in some cases, these values were statistically significantly higher. In some cases, age-related differences in the content of individual metals were also confirmed. Age-dependent increased Pb levels in the muscle and liver were reported in 6–7-month-old, <3–4-year-old, <5–6-year-old males. Similar relationships were confirmed for Zn in the bucks’ muscles. In the group of does, the increase in heavy metal concentrations with aging was observed for Zn in the muscle and for Pb in the liver. In the other cases, the trend of age-related level changes regarding heavy metals was not clearly demonstrated. Significant positive correlations between Cu and Cd were found for all samples, and significant negative correlations of Cu with Pb were found for the hair and liver. There was a negative correlation of Zn with Cu in all matrices. The study also revealed a negative correlation between Zn and Pb in muscle and a strong significant negative correlation between Zn and Cd in hair and in the liver.

**Supplementary Information:**

The online version contains supplementary material available at 10.1007/s11356-021-16425-6.

## Introduction

In toxicology, exposure to heavy metals is mainly measured by analysing the accumulation of these elements in the tissues and organs of living organisms. Therefore, these methods are strictly invasive. One exception is the hair, which can be sampled from a live animal in a non-invasive procedure without causing tissue injury or pain (Burger 2007; Gratacos-Cubarsi et al. 2006). Selected heavy metals, i.e. lead (Pb), cadmium (Cd), copper (Cu) and zinc (Zn), are natural elements of the Earth’s crust. As a result of human activity, including industrialization, their quantity in the environment is constantly increasing, producing negative impact on the biotic and abiotic components of the ecosystem (Neila et al. 2017; Pérez-López et al. 2016). These xenobiotics are defined as heavy metals due to their high atomic weights and high densities (Briffa et al. 2020). In the own research, four heavy metals with densities above 5 g/cm^3^, increasing in the Zn<Cd<Cu<Pb order, were investigated. They gain access to living organisms via a range of routes: respiratory, and gastrointestinal, i.e., with water and food. Considering the differences in the level of environmental pollution, the use of animal tissues for the monitoring of exposure to heavy metals seems to be a justified approach. Roe deer (*Caprelous capreolus* L.) is a species meeting a number of criteria which are required for sensitive bioindicator animals, because of the confirmed correlation between the level of environmental pollution and the degree of accumulation of toxic compounds in the tissues (Cygan-Szczegielniak et al. 2018; Garcia et al. 2011; Lehel et al., 2016; Pokorny and Ribaric-Lasnik 2002). Due to their strong territorial instinct and thus attachment to a specific area, these animals perfectly reflect the existing environmental conditions. Nevertheless, factors such as age, sex or sampling season may additionally influence the level of heavy metals in tissues (Pokorny and Ribaric-Lasnik 2002; Włostowski et al. 2006). In the case of sex, this may be due to differences in the toxicokinetics of these elements (Burger 2007; Legras et al. 2000). Pb can contaminate abiotic components of the ecosystem, e.g. soil and surface water bodies, and from there migrate to plants and accumulate in them. The next link in the food chain are animals and humans, and metals accumulate in their different tissues and organs, such as the liver, kidneys (Cai et al. 2009; Cygan-Szczegielniak 2021; Lehel et al. 2017), hair (Cygan-Szczegielniak 2021; Cygan-Szczegielniak et al. 2018; Kośla et al. 2011; Wongsasuluk et al. 2018) or muscles (Cygan-Szczegielniak 2021; Lehel et al. 2016; Lehel et al. 2017). Cd can easily be absorbed from the gastrointestinal tract, as well as cross the placental barrier and damage the structure of nucleic acid chains. It accumulates mainly in the kidneys and liver. Absorption of Cd also depends on factors such as exposure level, as well as the concentration of other coexisting elements (Włostowski et al. 2006). Pb and Cd are metals with a high toxicity factor. They can trigger various dysfunctions and cause acute and chronic poisoning in humans and animals. On the other hand, Zn and Cu are elements necessary for the health and growth of animals, and their toxicity may result from both deficiency and excessive exposure (Neila et al. 2017; Pérez-López et al. 2016). Zn is a component of regulatory proteins and DNA-binding proteins, while Cu is a component of many enzymes, including superoxide dismutase, tyrosinase, cytochrome c oxidase, and dopamine β-hydroxylase (Kim et al. 2019). Considering the persistence of heavy metals in the environment and their biomagnification in food chains, as well as their transfer from the environment to animal tissues, it is very difficult to assess the way of exposure to these elements in animals. Optimal proportions between elements at all stages of metabolism ensure that homeostasis important for animal health is maintained (French et al. 2017).

The aim of the study was to investigate the effects of age and sex on the content of heavy metals (Pb, Cd, Zn and Cu) in the hair, liver and the *longissimus lumborum* muscle of roe deer (*Capreolus capreolus* L.). In addition, correlations between heavy metals in individual tested matrices were studied as well as an attempt was made to explain the reasons for their accumulation in specific research matrices.

## Materials and methods

### Study material

**Fig. 1:**
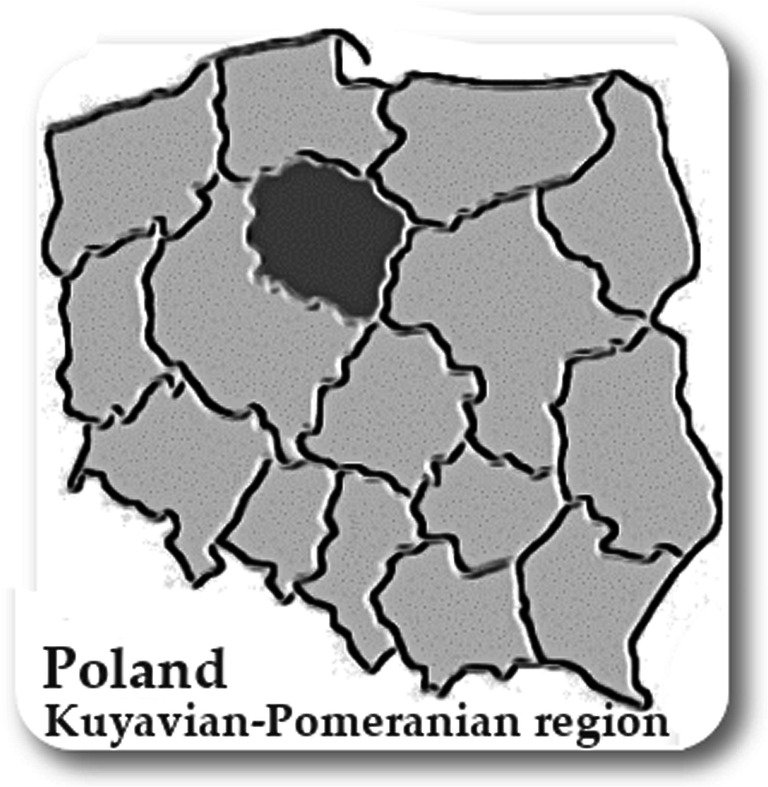
Map showing the hunting area in Poland (Kuyavian-Pomeranian region)

The study material comprised hair, liver and the muscle of roe deer (*Capreolus capreolus* L.) from the Kuyavian-Pomeranian province. The Kuyavian-Pomeranian province is a moderately industrialised region located in the central part of Poland (Fig. 1). The largest businesses in this region operate in the chemical industry, electrical and machinery industries, food processing industry, cellulose industry and printing services. Levels of metals measured in particulate matter (PM10) for which target values (e.g. cadmium, nickel and arsenic) or maximum acceptable limits (e.g. Pb) have been established were not exceeded at any of the monitoring stations. PM 10 particles are those of not more than 10 μm in diameter; they are a mixture of organic and inorganic compounds containing toxic substances including heavy metals. The average Pb concentration from all stations was 0.0120 μg/m^3^, while the Cd levels ranged from 0.4 to 0.5 ng/m^3^, with the target value of 5 ng/m^3^ (the report on the state of the environment Kuyavian-Pomeranian Province in the year 2017). The study comprised 56 animals: 28 females and 28 males in the following experimental setting: 6–7-month-old fawns ♀ (*n*=8), 3–4-year-old does (*n*=10) and 5–6-year-old does (*n*=10); and 6–7-month-old fawns ♂ (*n*=8), 3–4-year-old bucks (*n*=10) and 5–6-year-old bucks (*n*=10). The age of the animals was estimated based on the wear of selected teeth (Pérez-Barbería et al. 2014). Animals were obtained from their natural habitat, where the quantitative and qualitative composition of the vegetation eaten by them was impossible to control, and therefore, the diet of roe deer was not analysed for chemical composition.

The animals were shot by hunters in hunting districts within the Kuyavian-Pomeranian region in compliance with the Regulation of the Ministry of Environment 2005 (Journal of Laws, No. 48, item 459). According to this Regulation, bucks were shot between 11 May and 30 September, and does and fawns between early October and mid-January in the 2017/2018 hunting season.

### Sample preparation

Hair samples (*n*=56) were collected directly behind the *arcus costalis* in a site within approx. 40–50 cm from the gunshot wound to minimise the risk of sample contamination. Hair samples were dissected close to the skin from a 10 × 10-cm area and kept in a sealed polyethylene bag in a dry and shaded place until further analysis. To remove dirt and grease, samples were washed with acetone and placed for 15 min in an ultrasonic cleaner. Samples were stored for 12 h. Acetone was removed by decantation, and then the hair was rinsed twice with distilled water and dried in an oven at a temperature below 50°C. The appropriate procedure for preparing the hair for analysis ensured that it was free from potential superficial chemical contamination.

Samples of the muscle (from the segment along the first three lumbar vertebrae) and liver were also collected for analysis. For soft tissues, the distance from the gunshot wound is difficult to determine (various matrices). A usual distance was longer than 50 cm and the samples were collected with special caution. The liver (*n*=56) and the muscle (*n*=56) were lyophilized in a Lyovac GT2 freeze dryer (Finn-Aqua). All samples were then wet-digested using an EthosPlus microwave digestion system (Milestone) with the option of ATC-300 automatic temperatures control, according to Polish Standard PN-EN 13805:^2014^. For this purpose, 0.20 g aliquots of all analysed samples were prepared and then treated with 6.25 cm^3^ of a mixture of 65% HNO_3_ and 30% H_2_O_2_ in a 4:1 volume ratio (v:v). Digestion time was 20 min. Within the first 10 min, the temperature was gradually increased to 190°C and then maintained at 190°C±5°C. After completed digestion samples were completely transferred to 25-ml volumetric flasks and the volume was adjusted with distilled water. The concentration of mineral elements in the hair, liver and the *longissimus lumborum* muscle was expressed in milligrams per kilogram of dry weight (d.w.).

### Sample analysis

The concentration of Zn and Cu was measured by means of atomic absorption spectroscopy (F-AAS) using a SOLAAR 969 (Unicam) at wavelengths 213.9 nm for Zn and 324.8 nm for Cu. The concentrations of Pb and Cd were determined using flameless electrothermal atomic absorption spectrometry (ET-AAS) on a SOLAAR 939 QZ with deuterium background correction, GF 90 graphite cuvette and FS-90 Plus autosampler (Unicam). The wavelengths for Pb and Cd measurements were 283.3 nm and 228.8, respectively. Analyses were conducted at a certified laboratory in compliance with procedures proposed by Chatt and Katz (1989), and the Polish Standard PN-EN-14084:^2004^.

For Pb and Cd calibration and control, ClinCal®-B Calibrator, ClinChek®-WB Control Level I, II, III of Recipe Chemicals + Instruments (Germany) and SeronormTM Trace Elements at various levels of SERO AS (Norway) were used as reference materials. These materials were applied for both the calibration curve and internal quality control (precision and trueness). The precision parameters within a number of series for measurements of the same Pb and Cd concentrations on subsequent days and under the same analytical conditions (reproducibility) and the accuracy of the method are summarised in Table 1 (in the Supplementary Materials). The limits of detection (LOD) and quantitation (LOQ) for Pb and Cd (within one series of measurements) are summarised in Table 2 (in the Supplementary Materials).

**Table 1:** Effect of age on the content of heavy metals (mg kg^−1^ of d.w.) in the muscle, hair and liver of male roe deer

Matrix	Heavy metals (mg·kg^−1^)	6–7m.o.♂			3–4 y.o. ♂			5–6 y.o. ♂		
		*n*=8			*n*=10			*n*=10		
		$\overline{x}$	Me	SD	$\overline{x}$	Me	SD	$\overline{x}$	Me	SD
Muscle	Pb	2.88^A^	2.95	0.922	4.92^B^	4.45	1.59	5.45^B^	5.15	1.11
	Cd	0.474^a^	0.475	0.108	0.404^a^	0.375	0.099	0.424^a^	0.41	0.122
	Zn	70.5^a^	70.8	9.56	74.5^b^	75.70	10.3	76.3^c^	77.10	11.6
	Cu	43.3^a^	43.4	1.35	37.3^b^	42.00	9.91	30.6^b^	28.00	10.2
Hair	Pb	1.37^a^	1.00	0.625	1.06^a^	0.50	0.483	0.556^b^	0.375	0.396
	Cd	0.056^a^	0.054	0.014	0.090^b^	0.096	0.031	0.046^a^	0.031	0.042
	Zn	169^a^	160.03	35.1	126^a^	120.93	25.5	381^b^	376.8	25.5
	Cu	12.9^a^	10.60	4.66	15.1^a^	17.08	4.53	11.8^a^	11.20	1.17
Liver	Pb	0.710^a^	0.63	0.499	1.33^b^	0.697	0.870	1.73^c^	1.47	1.29
	Cd	0.065^a^	0.065	0.051	0.108^b^	0.115	0.037	0.068^a^	0.037	0.017
	Zn	220^a^	208.03	45.6	164^b^	157.21	33.1	496^c^	489.84	24.1
	Cu	16.7^a^	13.78	6.05	19.7^b^	22.20	5.89	15.3^a^	14.56	1.52

Values marked with different letters in the same row differ significantly: lower case letters (*p*<0.05), upper case letters (*p*<0.01); *SD*, standard deviation; *Me*, Q_2_, *n*, number of animals

**Table 2:** Effect of age on the content of heavy metals (mg kg^−1^ of d.w.) in the muscle, hair and liver of female roe deer

Matrix	Heavy metals (mg kg^−1^)	6–7 m.o. ♀			3–4 y.o. ♀			5–6 y.o. ♀		
		*n*=8			*n*=10			*n*=10		
		$\overline{x}$	Me	SD	$\overline{x}$	Me	SD	$\overline{x}$	Me	SD
Muscle	Pb	5.99^a^	5.26	2.38	6.05^a^	6.16	1.41	5.99^a^	5.66	1.22
	Cd	0.521^a^	0.523	0.119	0.477^b^	0.478	0.073	0.432^b^	0.423	0.125
	Zn	84.6^a^	84.96	11.5	89.4^b^	90.84	12.4	91.5^b^	92.52	13.9
	Cu	45.9^a^	46.0	1.44	39.5^b^	44.52	10.5	32.5^c^	29.68	10.8
Hair	Pb	1.51^A^	1.10	0.928	1.16^B^	0.55	0.931	0.611^C^	0.412	0.435
	Cd	0.058^a^	0.055	0.014	0.092^b^	0.098	0.032	0.047^a^	0.032	0.043
	Zn	203^A^	192.04	42.1	152^B^	145.12	30.6	408^C^	395.6	96.4
	Cu	13.7^a^	11.24	4.93	16.0^b^	18.11	4.80	12.7^a^	12.32	1.13
Liver	Pb	0.770^a^	0.688	0.549	1.46^b^	0.693	0.056	1.91^c^	1.52	1.42
	Cd	0.056^a^	0.054	0.052	0.110^b^	0.118	0.038	0.069^a^	0.038	0.017
	Zn	264^A^	249.65	54.7	197^B^	188.66	39.8	531^C^	514.33	25.4
	Cu	17.8^a^	14.66	6.42	20.9^b^	23.54	6.24	16.5^a^	16.02	1.47

Values marked with different letters in the same row differ significantly: lower case letters (*p*<0.05), upper case letters (*p*<0.01); *SD*, standard deviation; *Me*, Q_2_; *n*, number of animals

For Zn and Cu measurements, standard materials were prepared based on Merck KGaA aqueous standard solutions: Zn standard solution Zn(NO_3_)_2_ in HNO_3_ 0.5 mol L^−1^ and Cu standard solution Cu(NO_3_)_2_ in HNO_3_ 0.5 mol L^−1^. Concentrations of standards for Zn were, respectively, 0.25, 0.5, 1.0 and 2.0 mg L^−1^, and for Cu were 0.025, 0.05, 0.1 and 0.25 mg L^−1^, in 6 repetitions for each concentration. Based on the absorbance values from several series of standard substance measurements, the RSD (%) parameters were calculated for the precision control. The RSD (%) values ranged from 0.1 to 0.2% and 0.2 to 0.4% for Zn and Cu, respectively. LOD for Zn and Cu was 1 μg L^−1^. Both calibration and within-laboratory control procedures were performed for each series of measurements. All concentrations of the heavy metals in the studied matrices that are presented in the paper exceeded the LOD values.

### Statistical analysis

Most statistics did not conform to the requirements for normal distribution (which was verified using the Shapiro-Wilk test) or requirements for the homogeneity of variance, which are necessary to use parametric tests. For this reason, the significance of differences between experimental groups was analysed with the non-parametric *U* Mann-Whitney test and the Kolmogorov-Smirnov test. The significance of differences between multiple independent samples (groups) was analysed with a non-parametric test (ANOVA), the Kruskal-Wallis test, the median test, and multiple comparison of mean ranks for all samples. Correlations between selected parameters were analysed using Spearman’s rank correlation coefficients. The interaction between age and sex in the context of their impact on the level of heavy metals in individual matrices was also analysed. Interaction were analysed in a 2 × 2 factorial design (ANOVA), including the effects of age and sex and the data has been log transformed. The obtained data were processed using Statistica 13.1 software.

## Results

Table 1 presents the content of heavy metals in the hair, liver and muscle of bucks in relation to their age. Obtained data were characterised by a large variation. Of all heavy metals analysed, the concentration of Zn (in the muscle) and Pb (in the muscle and liver) increased with the age of male roe deer in the following order: 6–7 months old, <3–4 years old, <5–6 years old (Table 1). Table 2 presents the content of heavy metals in the hair, liver and muscle of female roe deer in relation to their age. In the group of does, age-related growing trends were observed for Zn in the muscle and for Pb in the liver (Table 2). On the other hand, the concentration of Pb in the hair decreased with the age of animals, regardless of their sex (Tables 1 and 2). The highest level of Zn was measured in the liver, and depending on the age, it ranged from 70.5 (mg kg^−1^) in the muscle of 6–7-month-old fawns to as much as 531 (mg kg^−1^) in the liver of 5–6-year-old does. The levels of Cu ranged from 11.8 in the hair of the oldest bucks to 45.9 (mg kg^−1^) in the muscle of female fawns. The levels of Pb were from 0.710 (mg kg^−1^) in the liver of male fawns to 6.05 mg kg^−1^ in the muscle of 3–4-year-old does. The concentrations of Cd ranged from 0.046 (mg kg^−1^) in the hair of the oldest bucks to 0.521 (mg kg^−1^) in the muscle of female fawns.

Table 3 presents sex-related differences in the levels of heavy metals in the muscle, hair and liver of animals, as well as interaction coefficients for sex and age, and in the levels of these elements in individual biological matrices. Even though in females, higher concentrations of heavy metals were noted in almost all study matrices, only in some cases, these values were statistically significantly higher. The only exception was Cd higher, but no statistically significant, concentrations of this metal were found in liver samples of 6- to 7-month-old male fawns compared to its levels in 6- to 7-month-old female fawns. As for the heavy metals considered, regardless of age, for females, a statistically significant higher content of the compounds was confirmed in 50% of the results obtained in the muscle and liver. In the case of hair, a higher and statistically significant content was reported for 33% of the results obtained in the female group. Details on the values of the elements and the interactions for sex and age are presented in Table 3. Only for some elements, statistically significant interactions were confirmed in individual study matrices depending on age and sex in the muscle of the youngest animals for Pb, Zn and Cu, for Pb and Zn in the group of 3–4-year-old animals and for Zn in 5–6-year-old female roe deer. On the other hand, in hair of 6–7-month-old roe deer, a statistically significant interaction was confirmed for Pb and Zn, and exclusively for Zn in 3–4-year-old animals and the oldest individuals. In the liver, a statistically significant interaction was found between age and sex for Zn and Cu in the youngest individuals and for Zn in 3–4-year-old individuals, also for Pb, Zn and Cu in the oldest individuals.

**Table 3:** Sex-related differences in the levels of heavy metals in the muscle, hair and liver from different age groups and the interaction coefficient (A&S) for age and sex

Matrix	Age (A)	Heavy metals (mg kg^−1^)	Sex (S)				
			♂ *n*=28		♀ *n*=28		
			$\overline{x}$	SD	$\overline{x}$	SD	A&S
Muscle	6–7 m.o. *n*=8	Pb	2.88^A^	0.922	5.99^B^	2.38	**
		Cd	0.474^a^	0.108	0.521^a^	0.119	NS
		Zn	70.5^a^	9.56	84.6^b^	11.5	*
		Cu	43.3^a^	1.35	45.9^b^	1.44	*
	3–4 y.o. *n*=10	Pb	4.92^A^	1.59	6.05^B^	1.41	**
		Cd	0.404^a^	0.099	0.477^a^	0.073	NS
		Zn	74.5^a^	10.3	89.4^b^	12.4	*
		Cu	37.3^a^	9.91	39.5^a^	10.5	NS
	5–6 y.o. *n*=10	Pb	5.45^a^	1.11	5.99^a^	1.22	NS
		Cd	0.424^a^	0.122	0.432^a^	0.125	NS
		Zn	76.3^a^	11.6	91.5^b^	13.9	*
		Cu	30.6^a^	10.2	32.5^a^	10.8	NS
Hair	6–7 m.o. *n*=8	Pb	1.37^a^	1.02	1.51^b^	1.13	*
		Cd	0.056^a^	0.014	0.058^a^	0.014	NS
		Zn	169^A^	35.1	203^B^	42.1	**
		Cu	12.9^a^	4.66	13.7^a^	4.93	NS
	3–4 y.o. *n*=10	Pb	1.06^a^	1.48	1.16^a^	1.63	NS
		Cd	0.090^a^	0.031	0.092^a^	0.032	NS
		Zn	126^A^	25.5	152^B^	30.6	**
		Cu	15.1^a^	4.53	16.0^a^	4.80	NS
	5–6 y.o. *n*=10	Pb	0.556^a^	0.396	0.611^a^	0.435	NS
		Cd	0.046^a^	0.042	0.047^a^	0.043	NS
		Zn	381^a^	25.5	408^b^	96.4	*
		Cu	11.8^a^	1.17	12.7^a^	1.13	NS
Liver	6–7 m.o. *n*=8	Pb	0.760^a^	0.499	0.770^a^	0.549	NS
		Cd	0.066^a^	0.051	0.056^a^	0.052	NS
		Zn	220^A^	45.6	264^B^	54.7	**
		Cu	16.7^a^	6.05	17.8^b^	6.42	*
	3–4 y.o. *n*=10	Pb	1.33^a^	0.870	1.46^a^	0.056	NS
		Cd	0.108^a^	0.037	0.110^a^	0.038	NS
		Zn	164^a^	33.1	197^b^	39.8	*
		Cu	19.7^a^	5.89	20.9^a^	6.24	NS
	5–6 y.o. *n*=10	Pb	1.73^a^	1.29	1.91^b^	1.42	*
		Cd	0.068^a^	0.017	0.069^a^	0.017	NS
		Zn	496^a^	24.1	531^b^	25.4	*
		Cu	15.3^a^	1.52	16.5^b^	1.47	*

Values marked with different letters in the same row differ significantly; lower case letters (*p*<0.05), upper case letters (*p*<0.01); *SD*, standard deviation; *n*, number of animals; significance age (A) and sex (S): NS = *p* > 0.05; ^*^ *p* < 0.05;^**^ *p* <0.01

In order to investigate the complex relationship between elements, Tables 3–5 in the Supplementary information present correlations between selected metals in individual tissues without classification by sex and age (in the Supplementary Materials). In the case of hair, statistically significant negative relationships were noted between Pb and Cu (*r*_*xy*_ =−0.369), Cd and Zn (*r*_*xy*_ =−0.531), Zn and Cu (*r*_*xy*_ =−0.409) and a positive and statistically significant correlation between Cd and Cu (*r*_*xy*_ =0.478). Also in the liver, statistically significant relationships were found between the same elements, i.e. a negative correlation between Pb and Cu (*r*_*xy*_ =−0.399), Cd-Zn (*r*_*xy*_ =−0.543), Zn-Cu (*r*_*xy*_ =−0.469) and a positive statistically significant correlation between Cd and Cu (*r*_*xy*_ =0.486). In the muscle, there were two negative, statistically significant correlations: Pb-Zn (*r*_*xy*_ =−0.435) and Zn-Cu (*r*_*xy*_ =−0.404) and only one positive correlation Cd-Cu (*r*_*xy*_ =0.304).

## Discussion

Measurement of heavy metal concentrations in the tissues and organs of wild animals provides a range of valuable data on local pollution. For this reason tissues of wild animals are often used for bioindication (Curi et al. 2012). Factors such as age, sex, exposure time or sampling season may influence the content of heavy metals in these tissues (Demesko et al. 2018; Garcia et al. 2011; Pokorny and Ribaric-Lasnik 2002). Therefore, it seems reasonable to test the hypothesis stating that sex and age have a real effect on the content of Pb, Cd, Zn and Cu in the hair, liver and muscle of roe deer.

Garcia et al. (2011) reported an increasing age-related trend for concentrations of Cd and Pb in the liver, kidneys and muscle of roe deer. The only exception in their study was Zn, whose concentration was higher in younger animals. For comparison, in our study, age-dependent increased Pb levels in the muscle and liver and Zn levels in the muscle were reported in males (Table 1). In the group of does, the increase in heavy metal concentrations with aging was observed only for Zn in the muscle and for Pb in the liver (Table 2). In the other cases, the trend of age-related level changes regarding heavy metals was not clearly demonstrated. In our own research, no clear pattern confirming these relationships was obtained, as was shown in the studies by Garcia et al. (2011). Other research confirmed that hard tissue, including hair, may be an equally good measurement matrix to reflect a degree of heavy metal accumulation (Cygan-Szczegielniak 2021; Cygan-Szczegielniak et al. 2018). Studies conducted by other authors also confirm the use of hard tissue in bioindication techniques. Age-related increase in their concentration in the bones and teeth of roe deer, which was related to the longer exposure time, was reported by Demesko et al. (2018). The effect of age as a variable was only confirmed for Zn, while for other elements (e.g. Cu and Pb) the type of matrix in which these metals were analysed was more important (higher concentrations were found in teeth). Interestingly, the levels of Cu in teeth and bones (5.28 and 5.74 mg kg^−1^, respectively) reported by Demesko et al. (2018) were about half of those measured in our study for the hair. The levels of Pb in the teeth and bones of 5–6-year-old does were comparable to the levels measured in our study in the hair of same-age animals. On the other hand, the concentration of Pb in the hair decreased with the age of animals, regardless of their sex (Tables 1 and 2). This could be associated with the seasonal change of summer to winter coat, potentially manifested by a greater chronological variability regardless of age.

The liver is an important organ involved in the detoxification of the body. Heavy metals such as Pb or Cd can have a negative effect on liver function and cause damage to hepatocytes. The monitoring of heavy metal concentrations in the liver is used for the evaluation of exposure of wild animals to these pollutants and provides important information on the effects of various environmental factors on the animal body (Neila et al. 2017).

The effect of sex on the content of Pd, Cd and Zn in the liver, muscle and kidneys was also reported by Garcia et al. (2011). In the cited study, the levels of toxic metals in most of the analysed tissues were higher in female animals, which is consistent with the trend obtained in our findings. Only the levels of Cd in the liver were significantly higher in male animals than in females. French et al. (2017) found higher levels of Cd, Cu and Zn (form 1.3 to 1.5 times higher) in the liver of female red deer. In many cases, the levels of heavy metals measured in the tissues of animals of different age and sex varied considerably, but the statistical analysis finally did not confirm any significant effect of these factors on the investigated traits. For example, Hoffman et al. (2007) found a considerable variation in the levels of elements in springbok depending on age and sex, but there were no significant differences between individual study groups with respect to the levels in the *longissimum lumborum* muscle. The hepatic levels of Pb, Cu and Zn were higher in does, and the differences were statistically significant for Zn and Cu (except for individuals 3–4 years old) (Table 3). Higher levels of these elements in the liver of male wild boar compared to females were reported by Neila et al. (2017). However, the differences in the cited study were insignificant, and sex could not be regarded as a variable determining the concentration of elements. On the other hand, Lehel et al. (2017) confirmed the effect of sex on the levels of Cd and Pb in the liver, muscle and kidneys of roe deer and found that Cd concentrations were higher in all organs and muscle of male animals, while Pb concentrations were higher in the liver and kidneys of female animals. No significant sex-related differences in the levels of Pb in meat were reported by Lehel et al. (2017). The specific effect of sex on the absorption and metabolism of pollutants in the body may result from the differences between females and males at almost all levels, including gene expression and biochemistry, physiology, morphology and ontogeny (Burger 2007; Legras et al. 2000). All these factors influence the traits of an individual, such as size, nutrition and hormonal metabolism, the level of uptake and toxicokinetics of various xenobiotics, and finally their impact on the body. In our own research, it demonstrated a higher tendency for accumulation of these compounds, which may be associated with the hormonal and enzymatic processes as well as enhanced absorption. Differences in toxicokinetics of heavy metals may also be associated with the amount and distribution of adipose tissue which is specifically related to the sex. Does usually have more adipose tissue and show a higher tendency for Pb accumulation as well as they are more sensitive to heavy metal effects (Burger 2007; Legras et al. 2000). Trace metals, such as Zn or Cu, are effectively controlled in the body, while xenobiotics, e.g. Pb or Cd, are not subjects of this control mechanism, and therefore, the risk of their harmful effects is potentially higher (Briffa et al. 2020). To predict internal concentrations of metals in animals given a specific environmental exposure, knowledge of the relationship between levels of xenobiotics and their toxicokinetics is necessary. Coefficients of assimilation and elimination of metals determine their accumulation in the body. Trace metals demonstrate similar toxicokinetics due to comparable physicochemical properties. For proper functioning of the body, a balance between Zn and Cu concentrations within specific physiological limits should be maintained. When the supply of these trace metals from various sources is higher, they should be effectively eliminated (Stępień and Bednarska 2015). The dynamics of these changes may be affected by various factors, including sex. In general, scientific literature reports contain very few data explaining the reasons for different sex-related concentrations of heavy metals as well as the intensity of toxicokinetic processes. To clearly confirm this relationship, future research on the subject should be undertaken, including other parameters such as enzymatic activity of carboxylesterase (CaE), glutathione S-transferase (GST) or metallothionein. The differences in sex-related accumulation of heavy metals may also be explained by another parameter, i.e. concentrations of metal-binding protein in the gonads which are higher in does compared to bucks (Legras et al. 2000). This may explain higher hepatic levels of metals in does observed in the own research.

In addition, apart from the factors considered in our study, the impact of the sampling season should not be ignored. Bucks are hunted in spring and summer, while does and fawns are hunted in autumn and winter, and this fact may also influence the obtained results. A study by Cygan-Szczegielniak et al. (2014) investigating the hair of cows revealed much higher levels of most heavy metals in samples collected in winter, and also confirmed the effect of sampling season on the degree of accumulation of these elements. Higher concentrations of these metals in the liver, muscle and hair of female roe deer could also be attributed to the higher levels of environmental pollution in autumn and winter due to the heating season and related emissions from domestic chimneys (The report on the state of the environment Kuyavian-Pomeranian Province in the year 2017).

In order to investigate the complex relationship between elements, Tables 3–5 in the Supplementary information present correlations between selected metals in individual tissues (in the Supplementary Materials). Similar to this study, negative interactions between Zn and Cu were reported for the hair of red deer by Cygan-Szczegielniak et al. (2018) and by Cygan-Szczegielniak (2021). A study conducted by Kabata-Pendias and Pendias (1999) also revealed a metabolically significant antagonism between Zn and Cu, and the competition between these elements for their absorption from the gastrointestinal tract. Neila et al. (2017) also reported a significant negative correlation between Cu and Zn levels in the liver of wild boar (*r*_*xy*_ =−0.201, *p*<0.05). In contrast to our study, Gasparik et al. (2012) found positive correlations between these elements in the muscles (*r*_*xy*_ = 0.59) and kidneys (*r*_*xy*_ = 0.40) of wild boar. Moreover, there is reciprocal competition between Cd and Zn in the animal body. Cd may lead to abnormal metabolism of Zn, which is a cofactor in many physiological reactions. Therefore, Cd may replace Zn in some important enzymes. Cd may displace Zn from metalloproteins, which leads to inhibited activity of zinc-containing enzymes (Kabata-Pendias and Pendias 1999). Cd also has a negative effect on the assimilation of Zn and may cause digestive disorders and apoptosis (Wajdzik et al. 2017). Pb has an antagonistic effect on Zn. Abnormally high concentrations of Pb in the body cause a decrease in the concentration of Zn, and this may trigger a number of disorders, for example, reduced concentration of superoxide dismutase (Bokara et al. 2008). A similar negative correlation was found in our study between Zn and Pb in the muscle (*r*_*xy*_ =−0.435).

## Conclusions

The study provided evidence regarding the effects of age and sex on the content of heavy metals in the tissues of roe deer. It can be concluded that for most analysed types of samples higher levels of heavy metals were found in the tissues of female roe deer but only in some cases, these values were statistically significantly higher. Age-related differences in the content of individual metals were also confirmed but the directions of changes were inconsistent. The conducted study and analytical data obtained from it confirmed that the hair, liver and muscles can be used as specific bioindicators for the measurement of heavy metal accumulation and can be very useful in studying differences in their concentration depending on age or sex and correlation. The study results can be a valuable source of information on the concentration of Pb, Cd, Zn and Cu in selected study matrices in roe deer, depending on age and sex. Although the results do not give an unambiguous answer as to the direction of these changes, they can be a reference point for further research in this area.

## Supplementary information

ESM 1 (DOCX 20 kb)

ESM 2 **(**DOCX 16.3 kb)

## Data Availability

The datasets used and analysed during the current study are available from the corresponding author on reasonable request.

## Declarations

The animals were shot by hunters in hunting districts within the Kuyavian-Pomeranian region in compliance with the Regulation of the Ministry of Environment of 16 March 2005 (Journal of Laws, No. 48, item 459). The research did not require additional approval from the ethics committee.
