# Supplementary material for: Effects of age and sex on the content of heavy metals in the hair, liver and the longissimus lumborum muscle of roe deer Capreolus capreolus L
Source: Environ Sci Pollut Res Int. 2021 Sep 16;29(7):10782–90. doi: 10.1007/s11356-021-16425-6 (PMC8783889; doi:10.1007/s11356-021-16425-6)
Supplement: Supplementary file 1 — (DOCX 20 kb) [file 11356_2021_16425_MOESM1_ESM.docx]

**Table 1** The precision parameters within a number of series for measurements of the same Pb and Cd concentrations on subsequent days and under the same analytical conditions (reproducibility) and accuracy of the method

| **Matrix** | **Heavy metal** | **Certified value** | **n** | **Determined value**  $\overline{x}$±SD | **Reproducibility,**  **CV (%)** | **Accuracy of the method**  **(%)** |
| --- | --- | --- | --- | --- | --- | --- |
| ClinChek®,  WB Control | Pb | 5.84 µg·dl^-1^ | 19 | 5.88±0.16 | 2.68 | 100.64 |
| ClinChek®,  WB Control | Cd | 1.17 µg·dl^-1^ | 19 | 1.22±0.06 | 4.97 | 104.27 |

**Table 2** The limits of detection (LOD) and quantitation (LOQ) for Pb and Cd (within one series of measurements)

| **Heavy metal** | **Determined value** | **LOD** | **LOQ** |
| --- | --- | --- | --- |
| Pb | 5.84 µg·dl^-1^ | 0.347 µg·dl^-1^ | 1.041 µg·dl^-1^ |
| Cd | 1.19 µg·dl^-1^ | 0.084 µg·dl^-1^ | 0.252 µg·dl^-1^ |
