# Supplementary material for: Effects of age and sex on the content of heavy metals in the hair, liver and the longissimus lumborum muscle of roe deer Capreolus capreolus L
Source: Environ Sci Pollut Res Int. 2021 Sep 16;29(7):10782–90. doi: 10.1007/s11356-021-16425-6 (PMC8783889; doi:10.1007/s11356-021-16425-6)
Supplement: Supplementary file 2 — (DOCX 16.3 kb) [file 11356_2021_16425_MOESM2_ESM.docx]

**Table 3** Correlation coefficients (r_xy_) for the levels of selected heavy metals in the muscle of roe deer

| Parameters  (mg·kg^-1^) | Cd | Zn | Cu |
| --- | --- | --- | --- |
| Pb | -0.063 | -0.435^*^ | -0.023 |
| Cd |  | 0.202 | 0.304^*^ |
| Zn |  |  | -0.404^*^ |

Correlation coefficients (r_xy_) significant at: p < 0.05 ^*^

**Table 4** Correlation coefficients (r_xy_) for the levels of selected heavy metals in the hair of roe deer

| Parameters  (mg·kg^-1^) | Cd | Zn | Cu |
| --- | --- | --- | --- |
| Pb | -0.015 | -0.158 | -0.369^*^ |
| Cd |  | -0.531^**^ | 0.478^*^ |
| Zn |  |  | -0.409^*^ |

Correlation coefficients (r_xy_) significant at: p < 0.05 ^*^; p < 0.01^**^

**Table 5** Correlation coefficients (r_xy_) for the levels of selected heavy metals in the liver of roe deer

| Parameters  (mg·kg^-1^) | Cd | Zn | Cu |
| --- | --- | --- | --- |
| Pb | -0.017 | -0.148 | -0.399^*^ |
| Cd |  | -0.543^**^ | 0.486^*^ |
| Zn |  |  | -0.469^*^ |

Correlation coefficients (r_xy_) significant at: p < 0.05 ^*^; p < 0.01^**^
